# Content validity of the Scale for Assessing the Risk of Phlebitis in Hospitalized Adults

**DOI:** 10.1590/0034-7167-2025-0274

**Published:** 2026-10-02

**Authors:** Maryana da Silva Furlan, Antônio Fernandes Costa Lima

**Affiliations:** I Universidade Cesumar. Maringá, Paraná, Brazil; II Universidade de São Paulo. São Paulo, São Paulo, Brazil

**Keywords:** Validation Studies, Risk Factors, Phlebitis, Inpatients, Patient Safety., Estudios de Validación, Factores de Riesgo, Flebitis, Pacientes Internos, Seguridad del Paciente.

## Abstract

**Objectives:**

to construct and validate the content of a scale for assessing the risk of phlebitis in hospitalized adult patients using peripheral intravenous therapy.

**Methods:**

a methodological study, developed according to DeVellis, in four stages, from January to July 2024. For content validity analysis, Content Validity Ratio (CVR) was calculated, including items that reached a critical point of 0.667 (first round) and 0.538 (second round).

**Results:**

the scale contained 95 items, including those with a value above the critical point of the CVR. Items that received suggestions from experts and also presented a CVR above the critical point were redacted and retained. A scale containing 28 items, distributed across three dimensions, was constructed.

**Conclusions:**

the scale showed good measures of content validity and response process. Since it was assessed as easy to understand, it became a useful tool for use in the care of patients undergoing peripheral intravenous catheter therapy.

## RESUMEN

**Objetivos:**

construir y validar el contenido de una escala para evaluar el riesgo de flebitis en pacientes adultos hospitalizados que utilizan terapia intravenosa periférica.

**Métodos:**

estudio metodológico, desarrollado según la propuesta de DeVellis, en cuatro etapas, de enero a julio de 2024. Para el análisis de validez de contenido, se calculó el *Content Validity Ratio* (CVR), incluyendo los ítems que alcanzaron un punto crítico de 0,667 (primera ronda) y 0,538 (segunda ronda).

**Resultados:**

la escala contenía 95 ítems, incluyendo aquellos con un valor superior al punto crítico del CVR. Los ítems que recibieron sugerencias de expertos y que además presentaron un CVR superior al punto crítico fueron eliminados y conservados. Se construyó una escala de 28 ítems, distribuidos en tres dimensiones.

**Conclusiones:**

la escala mostró buenas medidas de validez de contenido y proceso de respuesta. Al ser evaluada como de fácil comprensión, se convirtió en una herramienta útil para la atención de pacientes sometidos a terapia con catéter intravenoso periférico.

## INTRODUCTION

In the healthcare field, proactive and systematic risk management has traditionally focused on the important role of patient safety. However, due to the value-based care movement, risk management has required healthcare institutions to adopt a broader perspective, encompassing operational, strategic, financial, human capital, legal, technological, and infrastructure and environmental risk areas^(1)^. Risk management aims to proactively identify and mitigate^(2)^ the occurrence of adverse events (AEs), defined as harm resulting from healthcare, as well as to reduce or eliminate risks through mitigation strategies^(3)^.

Patient safety consists of a set of activities that, through processes, procedures, behaviors, and environments, aims to reduce risks, minimizing the occurrence of preventable harm to patients^(4)^. By assessing the healthcare risks to which a patient is exposed, nursing professionals can establish a person-centered care plan involving patients and their family/loved ones.

Phlebitis is characterized as inflammation of a vein. It may be accompanied by pain, tenderness, erythema, edema, the presence of purulent exudate and/or a palpable venous cord. Its etiology may be chemical, mechanical, infectious, or post-infusion, and its rapid identification is fundamental to directing the appropriate intervention^(5)^. The literature shows that risk factors for phlebitis are multifactorial^(6)^, including patient length of stay, antibiotic use, duration of peripheral intravenous catheter (PIVC) insertion, multiple catheter insertions, presence of comorbidities, use of Teflon^®^ catheters, patient vein quality, and unsuccessful PIVC insertion^(6)^. Therefore, the authors considered that these factors should be included in the construction of a scale for assessing the risk of phlebitis. From this perspective, the development and content validity of an instrument for this purpose has the potential to support nurses in prescribing care, inform prevention protocols, reduce the incidence of this AE, and optimize the use of healthcare resources and associated costs.

In addition to the structuring milestones of patient safety, such as the institution of the Brazilian National Patient Safety Program^(3)^, through Ordinance 529/2013 and Collegiate Board Resolution 36/2013, which establishes actions to promote patient safety and improve the quality of healthcare services^(7)^, the Brazilian National Health Regulatory Agency provides recommendations for safe intravenous device management, with emphasis on AE prevention. This document compiles safe practices for preventing incidents related to PIVC, including standardization of insertion, maintenance, monitoring, and recording^(8)^.

Although there are 71 scales in the world aimed at identifying the presence and severity of phlebitis, only three have psychometric assessment^(9)^, and these instruments were not developed for risk prediction. In Brazil, validated scales remain focused on site assessment and complications^(10,11)^, without considering predictive stratification. In parallel, the literature has advanced in identifying predictors and developing predictive models using machine learning^(12,13)^, which addresses the need for an applicable clinical instrument. Thus, the lack of a predictive scale with proven validity to predict the risk of phlebitis in hospitalized adults still persists.

The previously cited integrative review, which synthesizes the risk factors for phlebitis, highlights the relevance of a risk assessment scale applied to hospitalized adult patients to support nurses in clinical practice^(6)^. In this regard, the present study aligns with the results identified in this review, supporting the relevance of developing and validating a predictive instrument to support phlebitis prevention^(6)^.

From this perspective, the present study specifically emphasizes the early identification of the risk of phlebitis, which can favor care appropriately directed to patients’ individual needs. Therefore, knowing the risk factors in patients with PIVC lines makes it possible to avoid complications related to the use of this device during intravenous therapy (IVT), contributing to the efficiency of the care provided^(6)^. It is worth noting that phlebitis is a preventable AE and that there are strategies that can be implemented to reduce its occurrence and, consequently, contribute to reducing the costs associated with it.

PIVC failure is a significant problem worldwide, affecting one in three catheters. Therefore, healthcare system efforts are needed to address infection prevention and PIVC-related failure, as well as the sequels of treatment interruption, increased healthcare costs, and adverse patient outcomes^(14)^. These costs can range from US$3,000 to US$56,000 per episode for treatment and extend hospital stays from seven to 14 days^(15)^.

In Brazil, the report on incidents related to healthcare, between January and December 2022, showed that notifications involving PIVC constituted the third most reported incident (50,000 notifications), mostly from hospitals^(16)^. From January to December 2023, these notifications corresponded to the second most reported incident (65,000 notifications)^(17)^. In the state of São Paulo, between August 2023 and July 2024, incidents related to PIVC ranked first, with more than ten thousand notifications^(18)^.

It is important to emphasize that the selection and management of PIVC should be based on the most recent recommendations, guidelines, and evidence for the safe and effective treatment of patients who require it during hospitalization^(19)^. Therefore, PIVC care requires that nursing professionals are aware of the associated risks, prioritizing preventive activities that can mitigate the occurrence of AEs such as phlebitis (the main negative outcome), infiltration, extravasation, and catheter obstruction^(20)^.

Given the nursing team’s responsibility in IVT, the Regional Nursing Council of the State of São Paulo recommends the development of care protocols for drug infusion in IVT, ensuring standardized practices and patient safety. In this context, nurses lead patient care management in IVT based on technical and scientific knowledge for decision-making, clinical assessment of the venous network, device selection, and continuous patient monitoring, in accordance with the Nursing Process. Therefore, the existence of institutional protocols and periodic team training enhances quality of care, reduces risks and harm, and strengthens safety in IVT^(21)^.

IVT is one of the most frequently performed activities by the nursing team in the healthcare of hospitalized patients, making it essential to advance both in the early detection of the risk of phlebitis and in the implementation of more efficient and effective care^(5)^.

In the literature, there is no scale with evidence of validity aimed at predicting the risk of hospitalized adult patients developing phlebitis; the available scales aim to support the assessment of the presence and severity of phlebitis^(6)^. Therefore, the construction of a reliable scale, with evidence of validity, is an innovation for the continuous improvement of patient safety in IVT, supporting the development of an individualized and patient-centered care plan.

## OBJECTIVES

To construct and validate the content of a scale for assessing the risk of phlebitis in hospitalized adult patients using peripheral IVT.

## METHODS

### Ethical aspects

The study was approved by the Research Ethics Committees of the School of Nursing at the *Universidade de São Paulo* and the two co-participating hospitals, in accordance with Resolution 466 of December 12, 2012, which approves the guidelines and regulations for research involving human beings^(22)^.

### Study design, period, and location

This is a methodological study, which covered the development of a scale for assessing phlebitis risk, based on the model proposed by DeVellis^(23)^, from January to July 2024. Initially, the scale items were developed, and the measurement format was determined (January and February 2024). Then, the content was validated by a panel of judges (March to May 2024), and the response process with nurses was conducted (June to July 2024). It was based on the Standards for QUality Improvement Reporting Excellence^(24)^.

Data collection was carried out in two hospitals. The first is a large, private, philanthropic institution that offers highly complex care, including Adult and Pediatric Emergency Rooms, a surgical center, a Hemodynamics Unit, an Intensive Care Unit, hemodialysis, and Cardiology, Oncology, Infusion, and Diagnostic Imaging Centers, serving various medical specialties. It is accredited by the Joint Commission International. The second is a medium-complexity public teaching hospital, maintained by the *Universidade de São Paulo*, which offers medical, surgical, pediatric, and obstetric care, as well as an Intensive Care Unit, surgical center, and outpatient clinic.

### Sample, inclusion and exclusion criteria

To define and characterize the panel of experts, eligibility criteria were adopted based on Fehring’s framework^(25)^ for selecting experts in content validity studies, considering training, clinical experience, scientific production and/or involvement in research groups on the subject. The panel of judges was composed of healthcare professionals with clinical experience in PIVC, specialists in IVT, and researchers in the field of psychometrics, with a minimum of two years’ experience in research or participation in projects related to the topic, or in research groups on it.

Judge selection by convenience was carried out through simple searches on the *Lattes* Platform (lattes.cnpq.br) and the use of a national WhatsApp^®^ group composed of professionals with experience in IVT and vascular access. The terms “IVT”, “PIVC” and “phlebitis” were used on the *Lattes* Platform, followed by a screening of the curricula. The screening was based on reading the summary of each resume and publications from the last five years, selecting those who met the criteria for minimum experience and production/performance. An invitation to participate was sent via the contact email addresses publicly available in the selected resumes, explaining the study objectives, the participation criteria, the expected role of the evaluator, and the link to the assessment scale, with instructions regarding the deadline for feedback. In the national WhatsApp^®^ group, selection occurred through voluntary acceptance of the invitation and verification of the eligibility criteria provided in the characterization form. In total, 28 invitations were sent to form the panel of experts; 12 judges accepted to participate in the first round; and 13 judges accepted to participate in the second round of content validity.

An invitation letter, along with the Informed Consent Form (ICF), containing guidelines regarding the research objectives and data collection, was sent via email and WhatsApp^®^. Upon acceptance of participation through the signing of the ICF, the content validity phase began, during which experts completed the assessment via Google Forms^®^. This method allows for reaching populations, especially for conducting expert panels, geographically distributed across distinct regions^(26)^. Each round remained open for 30 days, with a reminder sent to participants on the 14^th^ day to maximize the return rate.

The response process included nurses working in the two aforementioned hospitals who had at least two years of experience and provided care to adult patients using PIVC for IVC. Nurses on vacation, leave, or absence during data collection were excluded. Of the 20 nurses invited, 15 agreed to participate in the study and five declined due to lack of time. Participating nurses received a link containing sociodemographic information (sex, age, professional experience, qualifications, and area of practice), study objectives, data collection guidelines, the ICF, and the scale assessment form.

### Study protocol

The manuscript resulted from the doctoral thesis “*Construção de uma diretriz assistencial para prevenção da ocorrência do evento adverso flebite em pacientes adultos hospitalizados*”, presented to the Graduate Program in Nursing Management at the School of Nursing of the *Universidade de São Paulo*. It describes the stages involved in constructing the items and their content validity: 1) Development of scale items: initially, the theoretical foundation of the construct consisted of conducting an integrative review^(6)^ carried out independently by two reviewers and consulting the Infusion Nurses Society (INS) on the risk factors for phlebitis described in the Standard of Practice in Nursing^(5)^, aiming to generate a set of items that reflected the purpose of the scale. Then, its dimensions were constructed, which were subsequently assessed by a panel of judges. The scale dimensions were organized from integrative review synthesis^(6)^, grouping the risk factors into three domains: patient-related factors, IVT-related factors, and procedure-related factors. The concept of risk for phlebitis was adopted as a characteristic attributed to an individual who presents a cluster of multiple risk factors related to patients’ characteristics, the IVT, and the procedure performed for PIVC insertion and maintenance;

2) Determining the measurement format: dichotomous variables (yes or no)^(23)^ were used to compose each item of the scale to assess the risk of developing phlebitis in hospitalized adult patients. The risk factors for phlebitis generated 95 items, which were submitted to the judges for assessment regarding the scale content;

3) Content validity by a panel of judges: content validity was assessed in two rounds, the first with 12 judges and the second with 13, with experts from all regions of Brazil invited. The Content Validity Ratio (CVR)^(27)^ was applied to analyze judges’ responses regarding item clarity, practical relevance, and theoretical relevance^(23)^. Clarity represents the degree to which an item is understandable, unambiguous, and uses language appropriate for the target audience. Practical relevance is the item’s suitability for clinical practice and its applicability in context. Theoretical relevance is the item’s alignment with the construct “risk of phlebitis” and its support in the literature. And comprehensiveness corresponds to the adequacy of the set of items to represent the proposed domains and cover the essential content of the construct^(23)^. Critical CVR values can be used to determine how many panel members need to agree on a key item and, therefore, which items should be included or discarded from the final scale. Items that reached a critical CVR of 0.667 in the first round and 0.538 in the second round were included^(27)^. A number of judges above ten was considered associated with acceptable reliability for assessing the scale^(28)^. Data was collected using a questionnaire via Google Forms^®^, containing instructions for completion and data regarding judge characterization (profession, qualifications, length of clinical experience with IVT/PIVC, area of expertise (clinical, teaching, and research), participation in research groups, and scientific production on the subject), in addition to the presentation of the scale items and their dimensions. The time spent completing the scale was timed and recorded by judges, who could include suggestions for improving the wording or risk factors not yet considered. The responses followed a dichotomous scale type. A score of 0 was used for “no” answers, and a score of 1 for “yes” answers; 4) Evidence of validity based on the response process refers to the degree of alignment between the construct to be measured and the cognitive processes triggered by respondents when interpreting items and selecting responses^(29)^, allowing verification of whether the scale version is clearly understood by the target population. A cognitive interview^(29)^ was adopted with nurses, with the aim of obtaining validity evidence regarding completion instruction comprehension, item content and wording, dichotomous response options, the relevance to clinical practice and the overall organization of the scale. Fifteen nurses, selected by convenience and working in the aforementioned hospitals, participated in the study, assessing each item of the scale version. Initially, they completed a sociodemographic characterization form (sex, age, professional experience, qualifications, and area of expertise) and then analyzed the clarity of the instrument via Google Forms^®^. Subsequently, they underwent a cognitive interview to further assess item comprehension and applicability and the scale’s structure.

### Analysis of results and statistics

In the integrative review^(6)^, the flowchart for identifying and selecting primary studies was presented in accordance with the Preferred Reporting Items for Systematic Reviews and Meta-Analyses recommendations^(30)^. The quantitative data obtained from the content validity and response process evidence were tabulated in Microsoft Excel^®^ for assessment using the R programming language^(31)^. In the content validity analysis process, the CVR was calculated, created by Lawshe and calculated using the formula CVR = (ne - N/2) / (N/2), where “ne” is the number of raters who positively judged the item, and “N” is the number of judges participating in the study^(27)^. The index has been indicated in the literature as not suffering from data inflation when the number of experts comprising the panel is adjusted^(27)^. Thus, the cut-off point was considered to be CVR 0.667 in the first round of judges’ assessment, and CVR 0.538^(27)^ in the second, with items with lower values being assessed, modified or excluded according to judges’ recommendations.

Participant sociodemographic characteristics were analyzed using descriptive statistics, through the frequency distribution of the investigated variables, with the results presented as percentages (%). For categorical variables, absolute and relative frequencies were calculated. When applicable, numerical variables were also described by measures of central tendency and dispersion (such as mean and standard deviation), according to the nature and distribution of the data.

The information obtained from the cognitive interview was analyzed using descriptive statistics, considering the following aspects: completion instruction comprehension; item content clarity; response option adequacy; overall organization of items; presence of difficulties in completing the questionnaire; and relevance of the scale for practical use. The results were presented as relative frequencies. Additionally, participants’ comments and observations were used to refine the scale. Whenever a nurse reported difficulty, doubt, or divergent interpretation regarding any item, the wording and/or response options were revised and adjusted to increase clarity and understanding by the target audience, strengthening the instrument’s applicability in clinical practice.

## RESULTS

Initially, an integrative review was conducted^(6)^, encompassing 31 primary quantitative studies. From this review, the following risk factors associated with the occurrence of phlebitis in hospitalized adult patients who used PIVC lines were obtained: length of hospital stay; hematological changes; number of medications administered; antibiotic use; PIVC line insertion duration; insufficient nursing care; multiple device insertions; presence of infection and comorbidities; pain at PIVC insertion site; use of a Teflon catheter; reduced patient mobility; family history of deep vein thrombosis; patient vein quality; skin elasticity; and unsuccessful insertion.

Therefore, 95 items were generated, constituting the initial version of the scale, which underwent content validity in two rounds: the first with 12 specialists (one physician and 11 nurses); and the second with 13 specialists (one physician and 12 nurses). The objective was to assess the adjustments made.

Judges from all regions of Brazil were invited to participate in the study. However, responses were only received from experts in the South, Southeast, and Northeast regions. The judges who participated in the first round were also invited to the second, and most of them participated in both.

In the first round, 66.6% of experts were female, with a mean age of 37.4 (SD=4.83) years. The mean professional experience was 14.1 (SD=5.18) years. The mean experience in IVT and/or psychometrics was nine (SD=5.92) years. Of these, 25% had a specialization, 50% a master’s degree, and 25% a doctoral degree. All worked in the healthcare field, 58.3% in teaching and research, and 50% had scientific publications in IVT, and 16.6% in psychometrics.

In the second round, 46.1% of specialists had participated in the first round of assessment, with 76.9% being female, with a mean age of 37.9 (SD=4.05) years. The mean experience time was 13.6 years (SD=4.40) and the mean experience time in IVT and/or psychometrics was nine years (SD=3.31). Of these, 30.7% had a specialization; had 46.1% a master’s degree; 15.3% a doctoral degree; and 7.6% a post-doctoral degree. All worked in care (92.3% in IVT), teaching (84.6% in IVT, and 7.6% in psychometrics) and research (69.2% in IVT, and 23% in psychometrics). Furthermore, 61.5% had scientific publications in IVT, and 15.3% in psychometrics.

In the first round of assessment by the 12 experts, a significance level of 95% was adopted, with a critical CVR of 0.667 in the first round and 0.538 in the second^(27)^. Items with values above the critical value of the CVR (relevant) were included in the scale. Items that had lower CVR in the clarity aspect were rewritten, respecting experts’ opinions. Items that had suggestions from the experts and presented CVR above the critical value^(27)^ were also changed (changes in writing and nomenclature) to make them more understandable to the scale’s target population.

The panel of experts highlighted the need for adjustments to the wording for greater clarity and a reduction in the number of items on the scale. In particular, they reiterated the difficulty nurses have in identifying drugs with the potential to damage the vascular system, which could compromise risk assessment in practice, emphasizing the importance of the institution providing a list of these drugs.

Among the 95 items presented in Chart 1, experts indicated the exclusion of 38 (2, 6-8, 12-14, 16, 18-24, 26, 35-39, 46, 5-52, 57, 68-69, 71-72, 75-77, 79, 82-84, 86 and 88). They suggested grouping or excluding items considered redundant, such as items 4 (“Patient with prolonged hospital stay for any reason?”) and 5 (“Patient with hospital stay longer than 6 days?”), which were unified and changed to “Patient with an expected duration of IVT longer than 6 days?”. It was recommended to exclude three items (9, 10, and 11), which would require a precise assessment, such as the use of ultrasound, and therefore could not be interpreted consistently by the professional in the context of routine use. Similarly, the exclusion of items that included the names of antibiotics and other medications was indicated, since the items that address the hydrogen potential (pH), the classification of the drug as an irritant or vesicant, and osmolarity already include them, reinforcing the use of the drug list to support the evaluator.

**Chart 1:** Items generated to compose the Scale for Assessing the Risk of Phlebitis in Hospitalized Adults, São Paulo, São Paulo, Brazil, 2024

Item
Female patient? - () no () yes
Patient aged between 19 and 48 years? - () no () yes
Patient older than 60 years? - () no () yes
Patient with prolonged hospital stay for any reason? - () no () yes
Patient with hospital stay longer than 6 days? - () no () yes
Patient with black skin color? - () no () yes
Patient with white skin color? - () no () yes
Patient with brown skin color? - () no () yes
Patient with regular vein quality? - () no () yes
Patient with poor vein quality? - () no () yes
Patient with veins difficult to visualize? - () no () yes
Patient with low skin elasticity? - () no () yes
Patient with family history of deep vein thrombosis? - () no () yes
Patient with immunodeficiency? - () no () yes
Patient with diabetes mellitus? - () no () yes
Patient with comorbidities? - () no () yes
Patient is malnourished? - () no () yes
Patient with current diagnosis of infection? - () no () yes
Patient with platelet count greater than 303,000? - () no () yes
Patient using an indwelling urinary catheter? - () no () yes
Patient with cranial disease? - () no () yes
Patient hospitalized in a neurology unit? - () no () yes
Patient in the postoperative period of neurosurgery? - () no () yes
Patient hospitalized in a surgical unit? - () no () yes
Patient with reduced mobility? - () no () yes
Patient is active? - () no () yes
Patient was punctured more than once for insertion of PIVC? - () no () yes
Patient required insertion of more than one PIVC during hospitalization to complete IVT? - () no () yes
Was skin antisepsis performed according to protocol before PIVC insertion? - () no () yes
Was the puncture performed in an emergency situation? - () no () yes
Is the proportion of PIVC relative to the vein size greater than or equal to 33.3%? - () no () yes
Patient using a PIVC made of Teflon? - () no () yes
Patient using a rigid (steel) PIVC? - () no () yes
Patient using a PIVC with an open system? - () no () yes
Patient using an in-line filter? - () no () yes
Patient using extension tubing as an accessory to PIVC? - () no () yes
Does PIVC have an integrated stabilization mechanism? - () no () yes
Was PIVC inserted into the vessel at an angle greater than 5°? - () no () yes
Was PIVC inserted by a professional with a university degree? - () no () yes
Patient using a PIVC with a 20G diameter? - () no () yes
Patient using a PIVC with an 18G diameter? - () no () yes
Is PIVC inserted on the dorsum of the hand? - () no () yes
Is PIVC inserted in the antecubital fossa? - () no () yes
Is PIVC inserted in the wrist joint? - () no () yes
Is PIVC located in a lower limb? - () no () yes
Is PIVC inserted on the patient’s dominant side? - () no () yes
Does the dressing have reinforced edges? - () no () yes
Is the dressing intact? - () no () yes
Does PIVC have a specific adhesive for stabilization? - () no () yes
Is the dressing used on PIVC sterile? - () no () yes
Has PIVC been inserted for more than 96 hours? - () no () yes
Is PIVC dwell time greater than 72 hours? - () no () yes
Were the connections disinfected with 70% alcohol before manipulation of PIVC? - () no () yes
Was hand hygiene performed for less than 10 seconds before manipulating PIVC? - () no () yes
Is the IVT infusion method continuous and intermittent? - () no () yes
Was flushing performed with normal saline before and after medication administration? - () no () yes
Does the patient have fewer than three medications prescribed via PIVC? - () no () yes
Patient using high-osmolarity medications? - () no () yes
Patient using parenteral nutrition with osmolarity <900 mOsm/L? - () no () yes
Do the drugs in use have osmolarity <600 mOsm/L? - () no () yes
Do the administered solutions have low pH (<5) or high pH (>9)? - () no () yes
Was contrast administered via PIVC? - () no () yes
Patient receiving intravenous amiodarone? - () no () yes
Patient receiving intravenous potassium chloride? - () no () yes
Does the parenteral nutrition contain more than 10% dextrose? - () no () yes
Patient using an irritant medication? - () no () yes
Patient using a vesicant medication? - () no () yes
Patient receiving isotonic fluid? - () no () yes
Patient using medications that act on blood and hematopoietic organs? - () no () yes
Patient using anti-inflammatory medication? - () no () yes
Patient using antifungal medication? - () no () yes
Patient receiving amino acid infusion via PIVC? - () no () yes
Patient using amphotericin? - () no () yes
Patient using flucloxacillin? - () no () yes
Patient using gastric mucosal protectors? - () no () yes
Patient using gastric antisecretory drugs? - () no () yes
Patient using tramadol hydrochloride? - () no () yes
Patient using oxacillin? - () no () yes
Patient using clarithromycin? - () no () yes
Patient using ceftriaxone? - () no () yes
Patient using intravenous amoxicillin-clavulanate? - () no () yes
Patient using intravenous sodium omeprazole? - () no () yes
Patient using levetiracetam via PIVC? - () no () yes
Patient using norepinephrine via PIVC? - () no () yes
Patient using nicardipine via PIVC? - () no () yes
Patient using nitroglycerin via PIVC? - () no () yes
Patient receiving intravenous antibiotics? - () no () yes
Did the patient fail to receive any care related to PIVC? - () no () yes
Was a prior assessment of PIVC insertion site performed? - () no () yes
Did the patient report pain at PIVC insertion site? - () no () yes
Was PIVC insertion site assessed once per shift? - () no () yes
Was the patient instructed to report pain and other symptoms related to PIVC? - () no () yes
Has PIVC remained unused for more than 24 hours? - () no () yes
Is there increased skin temperature near PIVC insertion site? - () no () yes
Is there a hematoma at PIVC insertion site? - () no () yes

*PIVC - peripheral intravenous catheter; IVT - intravenous therapy.*

After reviewing and adjusting 16 items that needed rewriting to improve clarity (CVR<0.667), the scale encompassed 35 items. In the second round of assessment, experts suggested new groupings, the exclusion of three items considered redundant (which did not reach the critical CVR value for practical relevance), and the rewriting of two items to improve clarity. At the end of the adjustments, the second version, called the Scale for Assessing the Risk of Phlebitis in Hospitalized Adults (EARFAH - *Escala para Avaliação de Risco de Flebite em Adultos Hospitalizados*), consisted of 28 items and three dimensions (patient-related factors, IV-related factors, and procedure-related factors), as shown in Chart 2.

**Chart 2:** Items included in the third version of the Scale for Assessing the Risk of Phlebitis in Hospitalized Adults after analysis by 25 specialists and feedback process with 15 nurses, São Paulo, São Paulo, Brazil, 2024

Dimensions	Items	Answer	
Patient-related factors	Female patient?	() No	() Yes
	Patient with an expected duration of IVT longer than 6 days?	() No	() Yes
	Patient older than 60 years?	() No	() Yes
	Patient with diabetes mellitus?	() No	() Yes
	Patient with reduced mobility? (“A person who, temporarily or permanently, has limited ability to interact with and use the environment. A person with reduced mobility includes individuals with disabilities, older adults, obese individuals, pregnant women, and patients with restrictions on leaving the bed”)	() No	() Yes
IVT-related factors	Was the patient punctured with more than one PIVC during hospitalization to complete IVT?	() No	() Yes
	Patient with prescription of a solution with osmolarity > 900 mOsm?	() No	() Yes
	Do the administered solutions have low pH (<5) or high pH (>9)?	() No	() Yes
	Solution containing more than 10% dextrose?	() No	() Yes
	Patient receiving an irritant medication? (solutions that cause burning, inflammation, and irritation of the endothelium)	() No	() Yes
	Patient receiving a vesicant medication? (solutions that cause blistering and tissue necrosis when extravasated)	() No	() Yes
	Is there evidence that pulsatile flushing with 0.9% normal saline was performed for PIVC maintenance?	() No	() Yes
	Did the patient report pain at the insertion site, around it, or along the course of PIVC vein?	() No	() Yes
	Was PIVC insertion site assessed once per shift and documented in the medical record?	() No	() Yes
	Was the patient instructed to report pain and other symptoms related to PIVC?	() No	() Yes
	Has PIVC remained unused for IVT for more than 24 hours?	() No	() Yes
Procedure-related factors	Is there increased skin temperature near PIVC insertion site?	() No	() Yes
	Is there a hematoma at PIVC insertion site?	() No	() Yes
	Is there hyperemia at PIVC insertion site?	() No	() Yes
	Patient with more than two puncture attempts for PIVC insertion?	() No	() Yes
	Is PIVC inserted at a joint site (e.g., wrist or antecubital fossa)?	() No	() Yes
	Is PIVC located in a lower limb?	() No	() Yes
	Is the dressing a sterile PIVC dressing (sterile, transparent, and semipermeable)?	() No	() Yes
	Is the dressing intact? (the dressing must not be wet, loose, or soiled)	() No	() Yes
	Is the patient using a needle-type PIVC (scalp)?	() No	() Yes
	Patient using a PIVC with extension tubing?	() No	() Yes
	Was the puncture performed in an emergency situation? (Emergency: health conditions implying intense suffering or imminent risk of death requiring immediate medical treatment)?	() No	() Yes
	Was PIVC insertion site assessed before medication administration?	() No	() Yes

*PIVC - peripheral intravenous catheter; IVT - intravenous therapy.*

Item clarity on EARFAH was assessed by 15 nurses (100%) working in medical-surgical clinics, orthopedics, general critical care units, oncology, Adult Intensive Care Units, and nursing supervision in the two hospitals included in the study. Of these, 86.6% were female, with a mean age of 32.8 years (SD=6.91); a mean professional experience of 8.13 years (SD=4.99); 80% had a specialization, 6.7% a master’s degree, and 13.3% a doctoral degree. The mean time to complete the EARFAH was 10 minutes (SD=5.70).

In the cognitive interview, regarding items 7, 8, 10, and 11 (Chart 2), nurses reported difficulty in identifying medications considered risk factors for phlebitis (irritants, vesicants, and those with extreme pH and high osmolarity). They indicated the need to provide, in the workplace, a list based on the institution’s standardized drugs containing medications with these characteristics, in order to support their rapid identification in daily care. For institutions that use electronic medical records, it was suggested to include a quick access link during the assessment and to implement an alert in the medical prescription when the drug constitutes a risk factor for phlebitis.

Nurses reported that EARFAH contributes to clinical practice by promoting autonomy in the assessment and identification of patients at risk of developing phlebitis, and that the response options (yes or no) are more feasible. Furthermore, the continuous assessment process and application of the instrument alerts nurses to the risks of IVT to which patients are exposed. All reported considering EARFAH understandable in terms of completion instructions, item content, response options, relevance to practice, and overall organization of items.

All participants (100%) reported understanding the scale, including the completion instructions, the content, and item wording. There was also consensus (100%) that the dichotomous response option is the most appropriate. Furthermore, 100% stated that the scale is relevant to clinical practice, approving of its overall organization. Although they pointed out some specific difficulties with the items related to drugs as risk factors, they did not report any other difficulties in completing the instrument.

## DISCUSSION

The first stage of this study synthesized knowledge regarding risk factors for the occurrence of phlebitis in hospitalized adult patients through the development of an integrative review^(6)^, which highlighted the risk factors associated with the development of this AE.

Based on the results of this integrative review^(6)^ and the use of INS recommendations^(5)^, 95 candidate items were generated for the first version of EARFAH which, after evidence of content validity and response process, was structured with three dimensions (patient-related factors, IVT-related factors and procedure-related factors) and 28 items. The concept of risk for phlebitis adopted was that of a characteristic attributed to the individual that presents the grouping of multiple risk factors related to patient characteristics, IVT and the procedure performed for PIVC insertion and maintenance.

Scales are developed to measure a phenomenon that cannot be directly quantified, but which is identified by a theory or concept as existing^(32)^. Risk factors for phlebitis are multifactorial, so it is necessary to assess the patient and identify which factors they present in order to establish an individualized care plan.

EARFAH was developed based on studies on the subject, as well as the knowledge of professionals with experience in research in psychometrics and IVT. Therefore, in addition to contributing to clinical nursing practice and patient safety, it fills a gap related to the lack of a validated scale for this purpose in hospital settings.

EARFAH was sent to specialists from all regions of the country, but despite the researcher’s efforts, the two panels held only included representatives from the South, Southeast, and Northeast regions. It is important to consider that the participation of specialists from other regions would contribute to adaptation in relation to the particularities of the language and the work context of the professionals who will use the scale^(33)^.

Evidence of content validity indicated which items needed corrections regarding clarity and which should be removed from the scale through an assessment of practical and theoretical relevance. Thus, the qualitative analysis by experts contributed to improvements in item wording, making them more understandable and suitable for practical use. The experts suggested creating a list for nurses of medications that are risk factors for the development of this AE, aiming to address difficulties in routine care.

The literature presents several medications that are risk factors that can result in vascular endothelial injury^(34-36)^. Therefore, it is essential that nurses know which medications pose a risk for phlebitis, as a lack of knowledge in identifying these drugs can lead to inadequate IVT planning, a lack of preventive care, and the development of complications^(5,8)^.

Although it is unlikely that nurses will know all the drugs capable of causing phlebitis, a support ecosystem with technology, documents that classify medications harmful to the venous network, and a collaborative partnership with the hospital pharmacy makes it possible to overcome the challenge of knowing these drugs. Incorporating such strategies into routine care not only increases the accuracy of applying EARFAH and consequently identifying the risk, but also strengthens the institutional culture of preventing AEs.

During professional training, topics such as pharmacology, the chemical properties of medications (osmolarity and pH), and peripheral venous catheterization promote safe care tailored to the needs of patients using IVT^(37)^. Therefore, recognizing medications that are risk factors will enable nurses to act proactively, preventing the occurrence of AEs. It is important to highlight that nurses with knowledge of medication-related risk factors are competent to establish preventive measures regarding the choice of IVT, and PIVC maintenance and monitoring^(38)^.

Some items from EARFAH were excluded based on the CVR analysis. According to experts, these were items that were not relevant to daily practice and/or could not be performed by nurses, such as the assessment of the ratio between the diameter of PIVC and the caliber of the vein in which it will be inserted, since this would require the use of an ultrasound device and the nurse would need to be properly trained for this assessment.

Technologies can be incorporated into best practices to provide a better experience for patients who require vascular access^(39)^. However, despite the advances, the lack of technology for PIVC insertion in most Brazilian hospitals is a reality and a challenge. Many healthcare services still face difficulties in obtaining and implementing the use of technologies such as ultrasound and venous network visualization devices. These devices are important to increase the success rate on the first puncture attempt, reduce complications and costs. Furthermore, it is emphasized that nurses need to be properly trained in their use.

Overall, the EARFAH content was assessed as relevant and clearly worded. The restructured and assessed items were in accordance with experts’ suggestions/guidelines, the nurses who participated in the response process, and the research team, as recommended in the literature^(23)^. Some items were adjusted to maintain the clarity and semantic appropriateness of EARFAH for the target population, helping to reduce the risks of bias in the interpretation of its content^(40)^.

The response process, despite being established for more than ten years^(41)^, is underutilized in the validity evidence of scales. Therefore, there is much to be explored. Regarding EARFAH clarity, in the response process stage, item 7 (“7. Patient with prescription of a solution with osmolarity > 900 mOsm?”) presented a CVR of 0.600 due to a lack of knowledge of medications that have this characteristic. Nurses reported that it was unclear how to assess this item and, like the specialists, also suggested including a list of medications with osmolarity > 900 mOsm for professionals to consult when applying the scale. The need for a nominal list of medications was emphasized, and, in the case of institutions using electronic medical records, this information should be included in the electronic medical prescription.

The nurses considered item 12 (“12. Is there evidence that pulsatile flushing with 0.9% normal saline was performed for PIVC maintenance?”) to be a difficult factor to analyze, as it requires prior assessment of the nursing record for its correct completion. This stage, due to the time and attention required by nurse, can represent a challenge in the application of the scale.

It is important to highlight that nursing records are necessary to substantiate the provision of care, as well as to provide data that support internal and external audit processes. Documenting the care provided by nursing staff is essential for preventing complications and ensuring continuity of care^(42)^. Thus, the use of complete and standardized records regarding the PIVC contributes to the quality of care and patient safety.

The nurses who participated in the response process stage reported that EARFAH contributes to clinical practice and promotes their autonomy in assessing and identifying patients at risk of developing phlebitis, in addition to considering the most viable response options (no or yes). Furthermore, they explained that the continuous assessment process and application of the scale alerts nurses to the risks of IV ingestion to which patients are exposed.

Finally, it should be emphasized that the stages of constructing and validating a scale are complex and require the collaborative work of various professionals. Everyone’s contribution, combined with the involvement of those who will use it, is essential for the availability of valid, reliable, easy-to-understand, and applicable scales^(43)^. It is corroborated that nurses have a duty to base their professional practice on the best available evidence^(44)^. Furthermore, care must be evidence-based, using validated scales^(19)^.

The use of EARFAH strengthens patient safety and improves nursing care, as it allows for the early identification of individuals at risk of phlebitis and guides evidence-based preventive decisions. Its systematic application standardizes risk assessment, reduces variability among professionals, and increases consistency of care. As a result, it tends to decrease the occurrence of phlebitis, contributing to cost reduction and safer care. Furthermore, it improves nursing records (objectivity, completeness, and auditability), promoting continuity of care. Therefore, the incorporation of EARFAH contributes to a patient safety culture, in line with recommendations from the World Health Organization and national guidelines.

### Study limitations

Limitations of this study include the use of a convenience sample, which may restrict the representativeness of the participants. Furthermore, the possibility of a response rate from invitees may have influenced the final composition of the respondent group, potentially leading to selection bias. Participant geographical distribution, concentrated in certain regions, is also a limitation, which may reduce the generalizability of the findings to other institutional contexts. Although content validity and the response process are relevant stages in the development of a scale, internal structure validity will be performed so that EARFAH can be applied to the intended population.

### Contributions to nursing, health, or public policy

The development and validity of EARFAH represent an advancement for clinical practice, identifying patients at risk of phlebitis and incorporating preventive measures. Obtaining a valid and accurate nursing and health measurement scale is essential for the safety and quality of care provided.

## CONCLUSIONS

Based on the integrative review and the contributions of experts who participated in two rounds of assessment, as well as nurses who worked in the response process, it was possible to construct the final version of EARFAH, composed of three dimensions and 28 items. This version presents evidence of content validity and response process validity, and is the first Brazilian scale to address this construct in hospital care.

EARFAH was well-received by the nurses interviewed during the response process, being considered an easy-to-understand scale. However, for its application in assessing the risk of phlebitis in hospitalized adult patients, further studies to assess other psychometric evidence should still be conducted.

## Data Availability

The research data are available within the article.
