# Supplementary material for: Content validity of the Scale for Assessing the Risk of Phlebitis in Hospitalized Adults
Source: Rev Bras Enferm. 2026 Oct 2;79:e20250274. doi: 10.1590/0034-7167-2025-0274 (PMC13631961; doi:10.1590/0034-7167-2025-0274)
Supplement: Translation

# Translation: Validade de conteúdo da Escala para Avaliação de Risco de Flebite em Adultos Hospitalizados

**DOI:** 10.1590/0034-7167-2025-0274pt

**Authors:** Maryana da Silva Furlan, Antônio Fernandes Costa Lima

**Affiliations:** I Universidade Cesumar. Maringá, Paraná, Brasil; II Universidade de São Paulo. São Paulo, São Paulo, Brasil

**Keywords:** Estudos de Validação, Fatores de Risco, Flebite, Pacientes Internados, Segurança do Paciente.

## Abstract

**Objetivos:**

construir e validar o conteúdo de escala para avaliação de risco de flebite em pacientes adultos hospitalizados utilizando terapia intravenosa periférica.

**Métodos:**

estudo metodológico, desenvolvido, conforme proposto por DeVellis, em quatro etapas, de janeiro a julho de 2024. Para análise de validade de conteúdo, calculou-se o *Content Validity Ratio* (CVR), incluindo-se os itens que atingiram o ponto crítico de 0,667, na primeira rodada, e 0,538, na segunda rodada.

**Resultados:**

a escala continha 95 itens, sendo incluídos aqueles com valor acima do ponto crítico do CVR. Os itens que receberam sugestões dos especialistas e também apresentaram CVR acima do ponto crítico foram redigidos e mantidos. Construiu-se escala contendo 28 itens, distribuídos em três dimensões.

**Conclusões:**

a escala apresentou boas medidas de validade de conteúdo e processo de resposta. Por ser avaliada como de fácil compreensão, tornou-se ferramenta útil para uso no cuidado aos pacientes em terapia via cateter intravenoso periférico.

## INTRODUÇÃO

Na área de saúde, o gerenciamento de riscos, de forma proativa e sistemática, está, tradicionalmente, direcionado ao importante papel da segurança do paciente. Contudo, devido ao movimento de cuidados baseados em valor, o gerenciamento de riscos tem exigido das instituições de saúde uma visão ampliada, abrangendo as áreas operacional, estratégica, financeira, de capital humano, jurídica, tecnológica e de riscos baseados em infraestrutura e ambiente^(1)^. A gestão de risco objetiva identificar e mitigar, de forma proativa^(2)^, a ocorrência de eventos adversos (EAs), definidos como danos decorrentes do cuidado à saúde, bem como reduzir ou eliminar os riscos por meio de estratégias de mitigação^(3)^.

A segurança do paciente consiste em um conjunto de atividades que, por meio de processos, procedimentos, comportamentos e ambientes, visa à redução de riscos, minimizando a ocorrência de dano evitável para o paciente^(4)^. Ao avaliar os riscos assistenciais aos quais o paciente está exposto, os profissionais de enfermagem poderão estabelecer um plano de cuidados centrado na pessoa envolvendo o paciente e seus familiares/entes queridos.

A flebite é caracterizada como a inflamação da veia. Ela pode ser acompanhada por dor, sensibilidade, eritema, edema, presença de exsudato purulento e/ou cordão venoso palpável. Sua etiologia pode ter origem química, mecânica, infecciosa ou pós-infusional, e a sua rápida identificação é fundamental para direcionar a intervenção apropriada^(5)^. A literatura evidencia que os fatores de risco para flebite são multifatoriais^(6)^, abrangendo o tempo de internação do paciente, o uso de antibióticos, o tempo de permanência do cateter intravenoso periférico (CIP), o cateter inserido várias vezes no paciente, a presença de comorbidades, o uso de cateter de Teflon^®^, a qualidade da veia do paciente e a inserção malsucedida do CIP^(6)^. Portanto, os autores consideraram que esses fatores deveriam compor a construção da escala para avaliação do risco de flebite. Nessa perspectiva, o desenvolvimento e a validação de conteúdo de um instrumento com essa finalidade têm potencial para apoiar o enfermeiro na prescrição de cuidados, subsidiar protocolos de prevenção, reduzir a incidência desse evento adverso, e otimizar o uso de recursos assistenciais e os custos associados.

No contexto brasileiro, além dos marcos estruturantes da segurança do paciente, como a instituição do Programa Nacional de Segurança do Paciente^(3)^, por intermédio da Portaria nº 529/2013 e da Resolução da Diretoria Colegiada n° 36/2013, que estabelece ações para a promoção da segurança do paciente e a melhoria da qualidade dos serviços de saúde^(7)^, a Agência Nacional de Vigilância Sanitária disponibiliza recomendações para o manejo seguro de dispositivos intravenosos, com ênfase na prevenção de EAs. Esse documento reúne práticas seguras para prevenção de incidentes relacionados ao CIP, incluindo padronização de inserção, manutenção, vigilância e registro^(8)^.

Apesar de existirem 71 escalas no mundo voltadas à identificação da presença e gravidade da flebite, apenas três apresentam avaliação psicométrica^(9)^, e tais instrumentos não foram desenvolvidos para a predição de risco. No Brasil, as escalas validadas permanecem direcionadas à avaliação do sítio e de complicações^(10,11)^, sem contemplar a estratificação preditora. Paralelamente, a literatura tem avançado na identificação de preditores e no desenvolvimento de modelos preditores com o uso de aprendizado de máquina^(12,13)^, fato que vai ao encontro da necessidade de um instrumento clínico aplicável. Dessa forma, ainda persiste a lacuna de uma escala preditora com validade comprovada para prever o risco de flebite em adultos hospitalizados.

A revisão integrativa, anteriormente citada, que sintetiza os fatores de risco para flebite, destaca a pertinência de uma escala de avaliação de risco aplicada a pacientes adultos hospitalizados para subsidiar o enfermeiro na prática clínica^(6)^. Nesse sentido, o presente estudo está alinhado aos resultados identificados nesta revisão, corroborando a relevância de desenvolver e validar um instrumento preditor para apoiar a prevenção da ocorrência de flebite^(6)^.

Nessa perspectiva, o presente estudo enfatiza, especificamente, a identificação precoce do risco para a ocorrência de flebite, a qual pode favorecer o cuidado adequadamente direcionado às necessidades individuais do paciente. Portanto, conhecer os fatores de risco em pacientes com CIP possibilita evitar complicações relacionadas ao uso desse dispositivo durante a terapia intravenosa (TIV), contribuindo para a eficiência do cuidado prestado^(6)^. Ressalta-se que a flebite é um EA evitável e que há estratégias que podem ser realizadas para reduzir a sua ocorrência e, consequentemente, contribuir à redução dos custos a ela associados.

A falha associada ao CIP é um problema mundialmente significativo, afetando um em cada três cateteres. Logo, são necessários esforços no sistema de saúde para abordar a prevenção da infecção e a falha decorrente do uso do CIP, além das sequelas da interrupção do tratamento, o aumento dos custos de saúde e os desfechos adversos para os pacientes^(14)^. Esses custos podem variar de US$ 3.000 a US$ 56.000 por episódio para tratamento e prolongar a internação hospitalar de sete a 14 dias^(15)^.

No Brasil, o relatório de incidentes relacionados à assistência à saúde, entre janeiro e dezembro de 2022, evidenciou que as notificações envolvendo CIP constituíram o terceiro incidente mais reportado (50.000 notificações), majoritariamente por hospitais^(16)^. De janeiro a dezembro de 2023, tais notificações corresponderam ao segundo incidente mais reportado (65.000 notificações)^(17)^. No estado de São Paulo, entre agosto de 2023 e julho de 2024, os incidentes relacionados ao CIP ocuparam o primeiro lugar, com mais de dez mil notificações^(18)^.

Ressalta-se que a seleção e o gerenciamento do CIP devem se basear nas mais recentes recomendações, diretrizes e evidências para o tratamento seguro e eficaz de pacientes que dele necessitam durante a hospitalização^(19)^. Então, os cuidados com o CIP requerem que os profissionais de enfermagem conheçam os riscos associados, priorizando atividades preventivas que possam mitigar a ocorrência de EAs como flebite (o principal desfecho negativo), infiltração, extravasamento e obstrução do cateter^(20)^.

Diante da responsabilidade da equipe de enfermagem na TIV, o Conselho Regional de Enfermagem do Estado de São Paulo recomenda a construção de protocolos assistenciais para a infusão de fármacos na TIV, assegurando a padronização de práticas e a segurança do paciente. Nesse contexto, o enfermeiro lidera o gerenciamento do cuidado do paciente em TIV a partir do conhecimento técnico-científico para a tomada de decisão, avaliação clínica da rede venosa, seleção do dispositivo e monitoramento contínuo do paciente, à luz do Processo de Enfermagem. Portanto, a existência de protocolos institucionais e de treinamento periódico da equipe potencializa a qualidade assistencial, reduz riscos, e danos e fortalece a segurança na TIV^(21)^.

A TIV é uma das atividades mais realizadas pela equipe de enfermagem na assistência à saúde de pacientes hospitalizados, sendo imprescindível avançar tanto na detecção precoce do risco da ocorrência de flebite quanto na implementação de cuidados mais eficientes e efetivos^(5)^.

Na literatura, não há uma escala com evidências de validade voltada à predição do risco de pacientes adultos hospitalizados desenvolverem flebite; as escalas disponíveis se propõem a subsidiar a avaliação da presença e da gravidade da flebite^(6)^. Portanto, a construção de uma escala confiável, com evidências de validade, é uma inovação para a melhoria contínua da segurança do paciente em TIV, subsidiando a elaboração de um plano de cuidados individualizados e centrado no paciente.

## OBJETIVOS

Construir e validar o conteúdo de escala para avaliação de risco de flebite em pacientes adultos hospitalizados em uso de TIV periférica.

## MÉTODOS

### Aspectos éticos

O estudo foi aprovado pelos Comitês de Ética em Pesquisa da Escola de Enfermagem da Universidade de São Paulo e dos dois hospitais coparticipantes, atendendo à Resolução n^o^ 466, de 12 de dezembro de 2012, que aprova as diretrizes e normas regulamentadoras de pesquisas envolvendo seres humanos^(22)^.

### Desenho, período e local do estudo

Trata-se de estudo metodológico, que abrangeu o desenvolvimento da escala para avaliação de risco de flebite, fundamentado no modelo proposto por DeVellis^(23)^, de janeiro a julho de 2024. Incialmente, foram elaborados os itens da escala, determinando-se o formato de medida (janeiro e fevereiro de 2024). Em seguida, o conteúdo foi validado por um painel de juízes (março a maio de 2024), e o processo de resposta com enfermeiros foi conduzido (junho a julho de 2024). Baseou-se no *Standards for QUality Improvement Reporting Excellence* ^(24)^.

A coleta de dados foi realizada em dois hospitais. O primeiro é uma instituição filantrópica, de grande porte e privada, que oferece assistência de alta complexidade, dispõe de Pronto Atendimento Adulto e Pediátrico, centro cirúrgico, Unidade de Hemodinâmica, Unidade de Terapia Intensiva, hemodiálise e Centros de Cardiologia, Oncologia, Infusão e Diagnóstico por Imagem, atendendo a diversas especialidades médicas. É acreditado pela *Joint Commission International*. O segundo é um hospital escola público de média complexidade, mantido pela Universidade de São Paulo, que possui atendimentos de clínica médica, cirúrgica, pediátrica, obstétrica, Unidade de Terapia Intensiva, centro cirúrgico e ambulatório.

### Amostra, critérios de inclusão e exclusão

Para a definição e caracterização do painel de especialistas, adotaram-se critérios de elegibilidade baseados no referencial de Fehring^(25)^ para seleção de especialistas em estudos de validade de conteúdo, considerando formação, experiência clínica, produção científica e/ou atuação em grupos de pesquisa na temática. O painel de juízes foi composto a partir de profissionais de saúde com experiência clínica com CIP, especialistas em TIV e pesquisadores na área de psicometria, com experiência mínima de dois anos em pesquisas ou participação em projetos associados à temática, ou em grupos de pesquisa sobre ela.

A seleção dos juízes por conveniência ocorreu por meio de buscas simples na Plataforma *Lattes* (lattes.cnpq.br) e utilização de um grupo nacional de *WhatsApp* ^®^ composto por profissionais com atuação/experiência em TIV e acesso vascular. Na Plataforma *Lattes*, foram utilizados os termos “TIV”, “CIP” e “flebite”, com posterior triagem dos currículos. A triagem foi fundamentada na leitura do resumo de cada currículo e das publicações relativas aos últimos cinco anos, sendo selecionados aqueles que atendiam aos critérios de experiência mínima e de produção/atuação. Enviou-se um convite de participação, por meio dos endereços eletrônicos de contato disponibilizados publicamente nos currículos selecionados, explicitando os objetivos do estudo, os critérios de participação, o papel esperado do avaliador e o *link* de acesso à escala de avaliação, com orientações quanto ao prazo para devolutiva. No grupo nacional de *WhatsApp* ^®^, a seleção ocorreu por adesão voluntária ao convite e verificação dos critérios de elegibilidade informados no formulário de caracterização. Ao todo, foram enviados 28 convites para compor o painel de especialistas; 12 juízes aceitaram participar na primeira rodada; e 13 juízes aceitaram participar na segunda rodada de validação de conteúdo.

Encaminhou-se, por *e-mail* e *WhatsApp* ^®^, uma carta convite juntamente com o Termo de Consentimento Livre e Esclarecido (TCLE), contendo as orientações quanto aos objetivos da pesquisa e coleta de dados. Após o aceite de participação, mediante a assinatura do TCLE, iniciou-se a etapa de validade de conteúdo, quando os especialistas preencheram a avaliação via *Google Forms* ^®^. Essa modalidade possibilita o alcance de populações, principalmente para a condução de painéis de especialistas, geograficamente distribuídas em regiões distintas^(26)^. Cada rodada permaneceu aberta por 30 dias, com envio de lembrete aos participantes no 14º dia para maximizar a taxa de retorno.

Foram incluídos no processo de resposta enfermeiros atuantes nos dois hospitais supracitados, que possuíam, no mínimo, dois anos de experiência e prestavam cuidados a pacientes adultos em uso de CIP para TIV. Foram excluídos enfermeiros em período de férias, licença e afastamento durante a coleta. Dos 20 enfermeiros convidados, 15 aceitaram participar do estudo e cinco recusaram devido à indisponibilidade de tempo. Os enfermeiros participantes do processo de resposta receberam o *link* para acesso contendo informações sociodemográficas (sexo, idade, tempo de experiência profissional, titulação e área e atuação), objetivos do estudo, orientações para coleta de dados, o TCLE e o formulário de avaliação da escala.

### Protocolo do estudo

O manuscrito resultou da tese de doutorado “Construção de uma diretriz assistencial para prevenção da ocorrência do evento adverso flebite em pacientes adultos hospitalizados”, apresentada ao Programa de Pós-Graduação em Gerenciamento em Enfermagem da Escola de Enfermagem da Universidade de São Paulo. Nele, são descritas as etapas de construção dos itens e validade de conteúdo:

1) Elaboração dos itens da escala: inicialmente, a fundamentação teórica do construto consistiu na realização de revisão integrativa^(6)^ conduzida de forma independente por dois revisores e em consultas ao *Infusion Nurses Society* (INS) sobre os fatores de risco para flebite descritos no *Standard of Practice in Nursing* ^(5)^, visando gerar um conjunto de itens que refletissem o propósito da escala. Em seguida, construíram-se as suas dimensões, que, posteriormente, foram avaliadas pelo painel de juízes. As dimensões da escala foram organizadas a partir da síntese da revisão integrativa^(6)^, agrupando os fatores de risco em três domínios: fatores relacionados ao paciente, fatores relacionados à TIV e fatores relacionados ao procedimento. Adotou-se o conceito de risco para flebite como sendo uma característica atribuída ao indivíduo que apresenta o agrupamento de múltiplos fatores de risco que estão relacionados às características do paciente, da TIV e do procedimento realizado para inserção e manutenção do CIP;

2) Determinação do formato de medição: foram utilizadas variáveis dicotômicas (não ou sim)^(23)^ para compor cada item da escala para avaliar o risco de desenvolvimento de flebite em pacientes adultos hospitalizados. Os fatores de risco para flebite geraram 95 itens, que foram encaminhados para a avaliação dos juízes quanto ao conteúdo da escala;

3) Validação do conteúdo por um painel de juízes: a validade de conteúdo ocorreu em duas rodadas de avaliação, sendo a primeira com 12 juízes, e a segunda, com 13, tendo sido convidados especialistas de todas as regiões do Brasil. Aplicou-se o *Content Validity Ratio* (CVR)^(27)^ para analisar a resposta dos juízes quanto à clareza dos itens, pertinência prática e relevância teórica^(23)^. A clareza representa o grau em que o item é compreensível, sem ambiguidade e com linguagem adequada ao público-alvo. A pertinência prática é a adequação do item à prática clínica e possibilidade de aplicação no contexto. A relevância teórica é o alinhamento do item ao construto “risco de flebite” e sustentação na literatura. E a abrangência corresponde à adequação do conjunto de itens para representar os domínios propostos e cobrir o conteúdo essencial do construto^(23)^. Os valores críticos do CVR podem ser usados para determinar quantos membros do painel precisam concordar com um item essencial e, portanto, quais itens devem ser incluídos ou descartados da escala final. Foram incluídos os itens que atingiram o ponto crítico do CVR de 0,667, na primeira rodada, e de 0,538, na segunda rodada^(27)^. Considerou-se um número acima de dez juízes como associado a uma confiabilidade aceitável para a avaliação da escala^(28)^. Realizou-se a coleta de dados por meio de questionário, via aplicativo *Google Forms* ^®^, contendo instruções para o preenchimento e dados referentes à caracterização dos juízes (profissão, titulação, tempo de experiência clínica com TIV/CIP, área de atuação (assistencial, ensino e pesquisa), participação em grupos de pesquisa e produção científica na temática), além da apresentação dos itens da escala e de suas dimensões. O tempo despendido no preenchimento da escala foi cronometrado e registrado pelos juízes, que puderam incluir sugestões para melhorar a redação ou fatores de risco ainda não contemplados. As respostas seguiram o tipo de escala dicotômica. Utilizaram-se pontuação 0, para respostas “não”, e pontuação 1, para respostas “sim”;

4) As evidências de validade baseadas no processo de resposta se referem ao grau de alinhamento entre o construto que se pretende mensurar e os processos cognitivos acionados pelos respondentes ao interpretar os itens e selecionar respostas^(29)^, permitindo verificar se a versão da escala é compreendida de forma clara pela população-alvo. Adotou-se a entrevista cognitiva^(29)^ com os enfermeiros, com o objetivo de obter evidências de validade acerca da compreensão das instruções de preenchimento, do conteúdo e da redação dos itens, das opções dicotômicas de resposta, da pertinência para a prática clínica e da organização geral da escala. Participaram 15 enfermeiros, selecionados por conveniência e atuantes nos hospitais anteriormente mencionados, os quais avaliaram cada item da versão da escala. Inicialmente, responderam a um formulário de caracterização sociodemográfica (sexo, idade, tempo de experiência profissional, titulação e área de atuação) e, em seguida, analisaram a clareza do instrumento via *Google Forms* ^®^. Posteriormente, concederam entrevista cognitiva para aprofundar a avaliação da compreensão e aplicabilidade dos itens e da estrutura da escala.

### Análise dos resultados e estatística

Na revisão integrativa^(6)^, apresentou-se o fluxograma de identificação e seleção dos estudos primários de acordo com as recomendações do *Preferred Reporting Items for Systematic Reviews and Meta-Analyses* ^(30)^. Os dados quantitativos obtidos na evidência de validade de conteúdo e processo de resposta foram tabulados no programa *Microsoft Excel* ^®^ para avaliação via linguagem R de programação^(31)^. No processo de análise da validade de conteúdo, calculou-se o CVR, criado por Lawshe e calculado através da fórmula CVR = (ne - N/2) / (N/2), sendo que “ne” é o número de avaliadores que julgou de forma positiva o item, e “N” é a quantidade de juízes participantes do estudo^(27)^. O índice tem sido indicado pela literatura por não sofrer inflação dos dados com a adequação do número de especialistas que compõem o painel^(27)^. Assim, considerou-se como ponto de corte o CVR 0,667 na primeira rodada de avaliação dos juízes, e o CVR 0,538^(27)^, na segunda, sendo os itens com valores menores avaliados, modificados ou excluídos conforme as recomendações dos juízes.

A caracterização sociodemográfica dos participantes foi analisada por estatística descritiva, por meio da distribuição de frequências das variáveis investigadas, com apresentação dos resultados em porcentagem (%). Para as variáveis categóricas, foram calculadas as frequências absolutas e relativas. Quando aplicável, as variáveis numéricas também foram descritas por medidas de tendência central e dispersão (como média e desvio padrão), conforme a natureza e distribuição dos dados.

As informações obtidas na entrevista cognitiva foram analisadas por estatística descritiva, contemplando os seguintes aspectos: compreensão das instruções de preenchimento; clareza do conteúdo dos itens; adequação das opções de resposta; organização geral dos itens; presença de dificuldades para o preenchimento e pertinência da escala para uso na prática. Os resultados foram apresentados em frequências relativas. Adicionalmente, os comentários e observações dos participantes foram utilizados como subsídio para o refinamento da escala. Sempre que o enfermeiro relatou dificuldade, dúvida ou interpretação divergente em relação a algum item, o enunciado e/ou as opções de resposta foram revisados e ajustados, visando ampliar a clareza e a compreensão pelo público-alvo, fortalecendo a aplicabilidade do instrumento na prática clínica.

## RESULTADOS

Inicialmente, realizou-se a revisão integrativa^(6)^, abrangendo 31 estudos primários quantitativos. A partir dessa revisão, obtiveram-se os seguintes fatores de risco associados à ocorrência de flebite em pacientes adultos hospitalizados que utilizaram CIP: tempo de internação; alteração hematológica; número de medicamentos administrados; uso de antibióticos; tempo de permanência do CIP; recebimento de cuidados de enfermagem insuficientes; dispositivo inserido várias vezes; presença de infecção e comorbidades; dor no local de inserção do CIP; uso de cateter de teflon; mobilidade reduzida do paciente; histórico familiar de trombose venosa profunda; qualidade da veia do paciente; elasticidade da pele; e inserção malsucedida.

Então, foram gerados 95 itens constituintes da versão inicial da escala que passou pela etapa de validação de conteúdo em duas rodadas: a primeira, com 12 especialistas (um médico e 11 enfermeiros); e a segunda, com 13 especialistas (um médico e 12 enfermeiros). O objetivo era avaliar os ajustes realizados.

Foram convidados a participar do estudo juízes de todas as regiões do Brasil. No entanto, houve retorno apenas dos especialistas das regiões Sul, Sudeste e Nordeste. Os juízes integrantes da primeira rodada foram convidados também para a segunda, e a maioria deles participou de ambas.

Na primeira rodada, 66,6% dos especialistas eram do sexo feminino, com média de idade de 37,4 (DP=4,83) anos. O tempo médio de experiência profissional foi de 14,1 (DP= 5,18) anos. O tempo médio de experiência na área de TIV e/ou psicometria foi de nove (DP=5,92) anos. Desses, 25% tinham especialização, 50%, mestrado, e 25%, doutorado. Todos atuavam na área assistencial, 58,3% no ensino e pesquisa, e 50% possuíam produção científica na área de TIV, e 16,6%, em psicometria.

Na segunda rodada, 46,1% dos especialistas haviam participado da primeira rodada de avaliação, sendo 76,9% do sexo feminino, com média de idade de 37,9 (DP=4,05) anos. O tempo médio de experiência foi de 13,6 anos (DP=4,40) e o tempo médio de experiência na área de TIV e/ou psicometria, de nove anos (DP=3,31). Desses, 30,7% possuíam especialização, 46,1%, mestrado; 15,3%, doutorado; e 7,6%, pós-doutorado. Todos atuavam na assistência (92,3%, na área de TIV), no ensino (84,6%, na área de TIV, e 7,6%, na de psicometria) e na pesquisa (69,2%, na área de TIV, e 23%, na de psicometria). Além disso, 61,5% possuíam produção científica na área de TIV, e 15,3%, na de psicometria.

Na primeira rodada de avaliação dos 12 especialistas, adotou-se nível de significância de 95%, com CVR crítico de 0,667, na primeira rodada, e 0,538, na segunda^(27)^. Os itens com valor acima do valor crítico do CVR (relevantes) foram incluídos na escala. Os itens que tiveram CVR menor no aspecto clareza foram redigidos, respeitando-se a opinião dos especialistas. Os itens que tiveram sugestões dos especialistas e apresentaram CVR acima do valor crítico^(27)^ também foram alterados (mudanças na escrita e nomenclatura), para deixá-los mais compreensíveis à população-alvo da escala.

O painel de especialistas destacou a necessidade de ajustes na redação para maior compreensão e redução dos itens da escala. Em especial, reiteraram a dificuldade que os enfermeiros possuem para identificar os fármacos com potencial de dano à rede vascular, o que poderia comprometer a avaliação do risco na prática, destacando a importância de a instituição disponibilizar uma lista com esses fármacos.

Entre os 95 itens, apresentados no Quadro 1, os especialistas indicaram a exclusão de 38 (2, 6-8, 12-14, 16, 18-24, 26, 35-39, 46, 5-52, 57, 68-69, 71-72, 75-77, 79, 82-84, 86 e 88). Eles sugeriram o agrupamento ou exclusão de itens considerados redundantes, como os itens 4 (“Paciente com tempo de internação hospitalar prolongado por qualquer motivo?”) e 5 (“Paciente com tempo de internação superior a 6 dias?”), os quais foram unificados e alterados para “Paciente com previsão de internação em uso de TIV superior a 6 dias?”. Recomendou-se a exclusão de três itens (9, 10 e 11), que demandariam uma avaliação precisa, como o uso do ultrassom, e, portanto, não poderiam ser interpretados de maneira consistente pelo profissional no contexto de uso rotineiro. Da mesma forma, indicou-se a exclusão dos itens que contemplavam o nome de antibióticos e outros medicamentos, pois os itens que abordam o potencial de hidrogênio (pH), a classificação do fármaco como irritante ou vesicante e a osmolaridade já os incluem, reforçando o uso da lista de fármacos para apoiar o avaliador.

**Quadro 1:** Itens gerados para compor a Escala para Avaliação de Risco de Flebite em Adultos Hospitalizados, São Paulo, São Paulo, Brasil, 2024

Item
Paciente do sexo feminino? - () não () sim
Paciente com idade entre 19 a 48 anos? - () não () sim
Paciente com mais de 60 anos? - () não () sim
Paciente com tempo de internação hospitalar prolongado por qualquer motivo? - () não () sim
Paciente com tempo de internação superior a 6 dias? - () não () sim
Paciente com cor de pele negra? - () não () sim
Paciente com cor de pele branca? - () não () sim
Paciente com cor de pele parda? - () não () sim
Paciente com qualidade da veia regular? - () não () sim
Paciente com qualidade da veia ruim? - () não () sim
Paciente com veias de difícil visualização? - () não () sim
Paciente com pouca elasticidade da pele? - () não () sim
Paciente com história familiar de trombose venosa profunda? - () não () sim
Paciente com imunodeficiência? - () não () sim
Paciente com diabetes mellitus? - () não () sim
Paciente com comorbidades? - () não () sim
Paciente está desnutrido? - () não () sim
Paciente com diagnóstico atual de infecção? - () não () sim
Paciente com contagem de plaquetas superior a 303.000? - () não () sim
Paciente em uso de sonda vesical de demora? - () não () sim
Paciente com doença craniana? - () não () sim
Paciente está internado em clínica neurológica? - () não () sim
Paciente está no pós-operatório de neurocirurgia? - () não () sim
Paciente está hospitalizado em unidade cirúrgica? - () não () sim
Paciente possui mobilidade reduzida? - () não () sim
Paciente está ativo? - () não () sim
Paciente foi puncionado mais de uma vez para inserção do CIP? - () não () sim
Paciente necessitou da inserção de mais de um CIP durante a internação hospitalar para concluir a TIV? - () não () sim
A antissepsia da pele foi realizada conforme protocolo antes da punção do CIP? - () não () sim
A punção foi realizada em situação de emergência? - () não () sim
A proporção do CIP em relação ao tamanho da veia é maior ou igual a 33,3%? - () não () sim
Paciente em uso de CIP fabricado com teflon? - () não () sim
Paciente está utilizando CIP rígido (aço)? - () não () sim
Paciente em uso de CIP com sistema aberto? - () não () sim
Paciente em uso de filtro em linha? - () não () sim
Paciente em uso de tubo de extensão como acessório do CIP? - () não () sim
O CIP possui mecanismo de estabilização integrado? - () não () sim
O CIP foi inserido no vaso num ângulo >5°? - () não () sim
O CIP foi inserido por um profissional com graduação? - () não () sim
Paciente está utilizando CIP de diâmetro 20G? - () não () sim
Paciente está utilizando CIP de diâmetro 18G? - () não () sim
O CIP está inserido no dorso da mão? - () não () sim
O CIP está inserido na fossa antecubital? - () não () sim
O CIP está inserido na articulação do punho? - () não () sim
O CIP está localizado em membro inferior? - () não () sim
O CIP está inserido no lado dominante do paciente? - () não () sim
O curativo está com bordas reforçadas? - () não () sim
O curativo está integro? - () não () sim
O CIP está com adesivo específico para estabilização do CIP? - () não () sim
A cobertura utilizada no CIP é estéril? - () não () sim
O CIP está inserido há mais de 96h? - () não () sim
O tempo de permanência do CIP é superior a 72h? - () não () sim
Foi realizada a desinfecção das conexões com álcool 70% antes de manipulação do CIP? - () não () sim
O tempo de higienização das mãos antes de manipulação do CIP foi inferior a 10 segundos? - () não () sim
O método de infusão da ‡TIV é contínuo e intermitente? - () não () sim
Foi realizado *flushing* com soro fisiológico antes e após a administração dos medicamentos? - () não () sim
Paciente possui menos que três medicações prescritas via CIP? - () não () sim
Paciente em uso de medicamentos de alta osmolaridade? - () não () sim
Paciente em uso de nutrição parenteral com osmolaridade <900 mOsm/L? - () não () sim
Os fármacos em uso possuem osmolaridade <600 mOsm/L? - () não () sim
As soluções administradas possuem pH baixo (<5) ou alto (>9)? - () não () sim
Foi administrado contraste via CIP? - () não () sim
Paciente está recebendo amiodarona intravenosa? - () não () sim
Paciente está recebendo cloreto de potássio intravenoso? - () não () sim
A nutrição parenteral possui dextrose com mais de 10%? - () não () sim
Paciente em uso de medicamento irritante? - () não () sim
Paciente em uso de medicamento vesicante? - () não () sim
Paciente está recebendo fluído isotônico? - () não () sim
Paciente em uso de medicamentos que atuam no sangue e nos órgãos formadores de sangue? - () não () sim
Paciente em uso de anti-inflamatório? - () não () sim
Paciente em uso de antifúngico? - () não () sim
Paciente está recebendo infusão de aminoácidos via CIP? - () não () sim
Paciente em uso de anfotericina? - () não () sim
Paciente em uso de flucloxacilina? - () não () sim
Paciente em uso de protetores da mucosa gástrica? - () não () sim
Paciente em uso de antissecretores gástricos? - () não () sim
Paciente em uso de cloridrato de tramadol? - () não () sim
Paciente em de oxacilina? - () não () sim
Paciente em de claritromicina? - () não () sim
Paciente em uso de ceftriaxona? - () não () sim
Paciente em uso de amoxicilina-clavulanato de potássio intravenoso? - () não () sim
Paciente em uso de omeprazol sódico intravenoso? - () não () sim
Paciente em uso de levetiracetam pelo CIP? - () não () sim
Paciente em uso de noradrenalina pelo CIP? - () não () sim
Paciente em uso de nicardipina pelo CIP? - () não () sim
Paciente em uso de nitroglicerina pelo CIP? - () não () sim
O paciente está em uso de antibiótico intravenoso? - () não () sim
Paciente deixou de receber algum cuidado relacionado ao CIP? - () não () sim
Foi realizada a avaliação prévia do local de inserção do CIP? - () não () sim
Paciente relatou dor no local de inserção do CIP? - () não () sim
O local de inserção do CIP foi avaliado uma vez por turno? - () não () sim
Paciente foi orientado para comunicar a presença de dor e outros sintomas relacionados ao CIP? - () não () sim
Paciente está há mais de 24h sem utilizar o CIP? - () não () sim
Há aumento de temperatura da pele próximo ao local de inserção do CIP? - () não () sim
Há presença de hematoma no local de inserção do CIP? - () não () sim

*CIP - cateter intravenoso periférico; TIV - terapia intravenosa.*

Após a revisão e ajustes em 16 itens que necessitavam de reescrita para adequação em relação à clareza (CVR<0,667), a escala abrangeu 35 itens. Na segunda rodada de avaliação, os especialistas sugeriram novos agrupamentos, a exclusão de três itens considerados redundantes, que não atingiram o valor crítico do CVR na pertinência prática, e a reescrita de dois itens para adequação quanto à clareza. Ao final dos ajustes, a segunda versão, denominada Escala para Avaliação de Risco de Flebite em Adultos Hospitalizados (EARFAH), ficou composta por 28 itens e três dimensões (fatores relacionados ao paciente, fatores relacionados à TIV e fatores relacionados ao procedimento), conforme apresentado no Quadro 2.

**Quadro 2:** Itens constantes na terceira versão da Escala para Avaliação de Risco de Flebite em Adultos Hospitalizados após a análise dos 25 especialistas e processo de resposta com os 15 enfermeiros, São Paulo, São Paulo, Brasil, 2024

Dimensões	Itens	Resposta	
Fatores relacionados ao paciente	Paciente do sexo feminino?	() Não	() Sim
	Paciente com previsão de uso de terapia intravenosa superior a 6 dias?	() Não	() Sim
	Paciente com mais de 60 anos?	() Não	() Sim
	Paciente com diabetes mellitus?	() Não	() Sim
	Paciente possui mobilidade reduzida? (“Aquela que, temporária ou permanentemente, tem limitada sua capacidade de relacionar-se com o meio e de utilizá-lo. Entende-se por pessoa com mobilidade reduzida, a pessoa com deficiência, idosa, obesa, gestante, paciente com restrição para sair do leito”)	() Não	() Sim
Fatores relacionados à TIV	Paciente foi puncionado com mais de um CIP durante a internação hospitalar para concluir a TIV?	() Não	() Sim
	Paciente com prescrição de solução com osmolaridade > 900 mOsm?	() Não	() Sim
	As soluções administradas possuem pH baixo (<5) ou alto (>9)?	() Não	() Sim
	Solução que possui dextrose com mais de 10%?	() Não	() Sim
	Paciente em uso de medicamento irritante? (soluções que provocam ardor, inflamação e irritação do endotélio)	() Não	() Sim
	Paciente em uso de medicamento vesicante? (soluções que causam bolhas e necrose tissular quando extravasados)	() Não	() Sim
	Há evidência de realização do *flushing* pulsátil com SF 0,9% para manutenção do CIP?	() Não	() Sim
	Paciente relatou dor no local de inserção, ao redor e pela extensão da veia do CIP?	() Não	() Sim
	O local de inserção do CIP foi avaliado uma vez por turno e registrado em prontuário?	() Não	() Sim
	Paciente foi orientado para comunicar a presença de dor e outros sintomas relacionados ao CIP?	() Não	() Sim
	Paciente está há mais de 24h sem utilizar o CIP para terapia intravenosa?	() Não	() Sim
Fatores relacionados ao procedimento	Há aumento de temperatura da pele próximo ao local de inserção do CIP?	() Não	() Sim
	Há presença de hematoma no local de inserção do CIP?	() Não	() Sim
	Há presença de hiperemia no local de inserção do CIP?	() Não	() Sim
	Paciente com mais de duas tentativas de punção para inserção do CIP?	() Não	() Sim
	O CIP está inserido em local de articulação (exemplo: punho ou fossa antecubital)?	() Não	() Sim
	O CIP está localizado em membro inferior?	() Não	() Sim
	O curativo é estéril próprio para CIP (estéril, transparente e semipermeável)?	() Não	() Sim
	O curativo está integro? (o curativo não pode estar úmido, solto ou sujo)	() Não	() Sim
	Paciente está utilizando CIP agulhado (*scalp*)?	() Não	() Sim
	Paciente em uso de CIP com tubo extensor?	() Não	() Sim
	A punção foi realizada em situação de emergência? (emergência: condições de agravo a saúde que impliquem sofrimento intenso ou risco iminente de morte, exigindo tratamento médico imediato)?	() Não	() Sim
	Foi realizada a avaliação do local de inserção do CIP antes de administrar a medicação?	() Não	() Sim

*CIP - cateter intravenoso periférico; TIV - terapia intravenosa.*

A clareza dos itens constantes da EARFAH foi avaliada por 15 enfermeiros (100%) atuantes em clínica médico-cirúrgica, ortopedia, unidade crítica geral, oncologia, Unidade de Terapia Intensiva Adulto e supervisão de enfermagem nos dois hospitais campo de estudo. Desses, 86,6% eram do sexo feminino, com idade média de 32,8 anos (DP=6,91); tempo médio de experiência profissional de 8,13 anos (DP=4,99); 80% possuíam especialização, 6,7%, mestrado, e 13,3%, doutorado. O tempo médio para o preenchimento da EARFAH foi de 10 minutos (DP=5,70).

Na entrevista cognitiva, em relação aos itens 7, 8, 10 e 11 (Quadro 2), os enfermeiros relataram dificuldade para identificar os medicamentos considerados fatores de risco para flebite (irritantes, vesicantes e aqueles com extremos de pH e alta osmolaridade). Indicaram a necessidade de disponibilizar, no ambiente de trabalho, uma lista baseada nos fármacos padronizados pela instituição contendo os medicamentos com essas características, a fim de apoiar a sua rápida identificação no cotidiano assistencial. Para instituições que utilizam prontuário eletrônico, foi sugerida a inclusão de um *link* de acesso rápido durante a avaliação e a implementação de um alerta na prescrição médica quando o fármaco constituir fator de risco para flebite.

Os enfermeiros relataram que a EARFAH contribui com a prática clínica, promovendo autonomia na avaliação e identificação dos pacientes com risco de desenvolver a flebite e que as opções de resposta (não ou sim) são mais viáveis. Ademais, o processo de avaliação contínuo e aplicação do instrumento alerta o enfermeiro sobre os riscos da TIV que o paciente está exposto. Todos informaram considerar a EARFAH compreensível em relação às instruções de preenchimento, conteúdo dos itens, opções de respostas, pertinência na prática e organização geral dos itens adequadas.

Todas as participantes (100%) relataram compreender a escala, incluindo as instruções de preenchimento, o conteúdo e a redação dos itens. Também houve consenso (100%) de que a opção dicotômica de resposta é a mais adequada. Além disso, 100% afirmaram que a escala é pertinente para a prática clínica, aprovando sua organização geral. Apesar de apontarem dificuldade pontual nos itens relacionados aos fármacos como fatores de risco, não referiram outras dificuldades para o preenchimento do instrumento.

## DISCUSSÃO

A primeira etapa do presente estudo sintetizou o conhecimento relativo aos fatores de risco para a ocorrência do EA flebite em pacientes adultos hospitalizados por intermédio do desenvolvimento de revisão integrativa^(6)^, que evidenciou os fatores de risco associados ao desenvolvimento desse EA.

A partir dos resultados desta revisão integrativa^(6)^ e do uso das recomendações da INS^(5)^, foram gerados os 95 itens candidatos à primeira versão da EARFAH que, após as evidências de validade de conteúdo e processo de resposta, ficou estruturada com três dimensões (fatores relacionados ao paciente, fatores relacionados à TIV e fatores relacionados ao procedimento) e 28 itens. O conceito de risco para flebite adotado foi o de uma característica atribuída ao indivíduo que apresenta o agrupamento de múltiplos fatores de risco relacionados a características do paciente, da TIV e do procedimento realizado para inserção e manutenção do CIP.

As escalas são desenvolvidas para mensurar um fenômeno que não pode ser quantificado diretamente, mas que é apontado por uma teoria ou conceito como sendo existente^(32)^. Os fatores de risco para flebite são multifatoriais, por isso é necessário avaliar o paciente e identificar quais fatores que ele apresenta para estabelecer um plano de cuidados individualizado.

A EARFAH foi desenvolvida a partir de estudos sobre o tema, bem como do conhecimento de profissionais com experiência em pesquisa na área de psicometria e TIV. Portanto, além de contribuir para a prática clínica de enfermagem e a segurança do paciente, preenche uma lacuna relativa à inexistência de uma escala validada para essa finalidade no contexto hospitalar.

A EARFAH foi encaminhada para especialistas de todas as regiões do país, mas, apesar das tentativas da pesquisadora, os dois painéis realizados contaram apenas com representantes das regiões Sul, Sudeste e Nordeste. Importante considerar que a participação de especialistas de outras regiões contribuiria para a adaptação em relação às particularidades da linguagem e ao contexto de trabalho dos profissionais que utilizarão a escala^(33)^.

As evidências de validade de conteúdo indicaram os itens que precisaram de correções quanto à clareza e quais deveriam ser retirados da escala por meio da avaliação da pertinência prática e teórica. Dessa forma, a análise qualitativa dos especialistas contribuiu com os aprimoramentos na redação dos itens, tornando-os mais compreensíveis e adequados ao uso na prática. Os especialistas sugeriram construir, para os enfermeiros, uma lista com os fármacos que são fatores de risco para o desenvolvimento desse EA, visando sanar as dificuldades na rotina assistencial.

A literatura apresenta diversos medicamentos que consistem fatores de risco que podem resultar em lesão do endotélio vascular^(34-36)^. Portanto, é essencial que o enfermeiro conheça quais são os medicamentos com risco para ocorrências de flebite, pois a falta de conhecimento para a identificação desses fármacos pode levar ao planejamento inadequado da TIV, à falta de cuidados preventivos e ao desenvolvimento de complicações^(5,8)^.

Embora seja improvável que o enfermeiro conheça todos os fármacos capazes de causar flebite, um ecossistema de apoio com tecnologia, documentos que classificam os medicamentos danosos à rede venosa e uma parceria colaborativa com a farmácia hospitalar permite superar o desafio de conhecer esses fármacos. Incorporar tais estratégias à rotina assistencial não apenas aumenta a acurácia para aplicação da EARFAH e consequente identificação do risco, mas também fortalece a cultura institucional de prevenção da ocorrência de EAs.

Durante o processo de formação profissional, temas como farmacologia, propriedade química dos medicamentos (osmolaridade e pH) e cateterismo venoso periférico promovem um cuidado seguro de acordo com as necessidades do paciente em uso da TIV^(37)^. Logo, reconhecer os medicamentos que são fatores de risco possibilitará ao enfermeiro atuar de forma proativa, prevenindo a ocorrência de EA. Destaca-se que o enfermeiro com conhecimento sobre os fatores de riscos medicamentosos possui competência para estabelecer medidas de prevenção quanto à escolha do dispositivo para TIV, e manutenção e monitoramento do CIP^(38)^.

Alguns itens da EARFAH foram excluídos a partir da análise do CVR. Segundo os especialistas, tratavam-se de itens sem pertinência para a prática diária e/ou que não poderiam ser realizados pelo enfermeiro, como, por exemplo, a avaliação da proporção entre o diâmetro do CIP e o calibre da veia em que ele será inserido, pois seria necessário o uso do aparelho de ultrassonografia e o enfermeiro deveria estar devidamente capacitado para essa avaliação.

Tecnologias podem ser incorporadas às boas práticas para proporcionar uma melhor experiência aos pacientes que necessitam de acesso vascular^(39)^. Mas, apesar dos avanços, a ausência de tecnologia para a inserção do CIP na maioria dos hospitais brasileiros é uma realidade e um desafio. Muitos serviços de saúde ainda enfrentam dificuldades para obter e implementar o uso de tecnologias, como a ultrassonografia e os dispositivos de visualização da rede venosa. Esses equipamentos são importantes para aumentar a taxa de sucesso na primeira tentativa de punção, reduzir complicações e custos. Além disso, reforça-se que o enfermeiro precisa ser devidamente qualificado à utilização.

Em geral, o conteúdo da EARFAH foi avaliado como relevante e com abordagem clara. Os itens reestruturados e avaliados estiveram em concordância com as sugestões/orientações dos especialistas, dos enfermeiros que participaram do processo de resposta e da equipe de pesquisa, conforme preconiza a literatura^(23)^. Alguns itens foram ajustados para manter a clareza e a adequação semântica da EARFAH para a população-alvo, propiciando reduzir os riscos de viés de interpretabilidade do conteúdo^(40)^.

O processo de resposta, apesar de estar estabelecido há mais de dez anos^(41)^, é pouco utilizado nas evidências de validade das escalas. Logo, há muito a ser explorado. Em relação à clareza da EARFAH, na etapa do processo de resposta, o item 7 (“Paciente com prescrição de solução com osmolaridade >900mOsm?”) apresentou CVR 0,600 devido ao desconhecimento dos medicamentos que possuam essa característica. Os enfermeiros relataram que não estava claro como avaliar esse item e, assim como os especialistas, também sugeriram a inclusão da relação dos medicamentos com osmolaridade > 900 mOsm para consulta do profissional no momento da aplicação da escala. Foi enfatizada a necessidade de uma lista nominal dos medicamentos e, em caso de instituições que utilizam prontuário eletrônico, inserir essa informação na prescrição médica eletrônica.

Os enfermeiros consideraram um fator de difícil análise o item 12 (“Há evidência de realização do *flushing* pulsátil com SF 0,9% para manutenção do CIP?”), visto requerer a avaliação prévia do registro de enfermagem para seu correto preenchimento. Essa etapa, por demandar dispêndio de tempo e atenção do enfermeiro, pode representar um desafio na aplicação da escala.

Cabe destacar que os registros de enfermagem são necessários para fundamentar a prestação de cuidados, além de fornecer dados que subsidiam os processos de auditoria internas e externas. A documentação dos cuidados realizados por profissionais da equipe de enfermagem é essencial para a prevenção de complicações e continuidade do cuidado^(42)^. Assim, o uso de registro completo e padronizado sobre o CIP contribui para a qualidade assistencial e segurança do paciente.

Os enfermeiros que participaram da etapa do processo de resposta relataram que a EARFAH contribui para a prática clínica e promove sua autonomia na avaliação e identificação de pacientes com risco de desenvolver flebite, além de considerarem as opções de resposta (não ou sim) mais viáveis. Ademais, explicitaram que o processo de avaliação contínuo e aplicação da escala alerta o enfermeiro sobre os riscos da TIV aos quais o paciente está exposto.

Ressalta-se, por fim, que as etapas de construção e validação de uma escala são complexas e requerem o trabalho colaborativo de diversos profissionais. A contribuição de todos, aliada ao envolvimento daqueles que utilizarão, é imprescindível para a disponibilização de escalas válidas, confiáveis, de fácil compreensão e aplicabilidade^(43)^. Corrobora-se que o enfermeiro tem o dever de pautar sua prática profissional nas melhores evidências disponíveis^(44)^. Ademais, a assistência deve estar fundamentada em evidências, utilizando escalas validadas^(19)^.

O uso da EARFAH fortalece a segurança do paciente e qualifica a assistência de enfermagem, pois permite a identificação precoce de indivíduos com risco de flebite e a orientação de decisões preventivas baseadas em evidências. Sua aplicação sistematizada padroniza a avaliação de risco, reduz a variabilidade entre profissionais e aumenta a consistência do cuidado. Como resultado, tende a diminuir a ocorrência de flebite, contribuindo com a redução de custos e assistência segura. Além disso, qualifica os registros de enfermagem (objetividade, completude e auditabilidade), favorecendo a continuidade do cuidado. Logo, a incorporação da EARFAH contribui para a cultura de segurança do paciente, em consonância com recomendações da Organização Mundial da Saúde e diretrizes nacionais.

### Limitações do estudo

Como limitações do estudo, destaca-se a utilização de amostra por conveniência, o que pode restringir a representatividade dos participantes. Além disso, a possibilidade da taxa de retorno dos convidados pode ter influenciado a composição final do grupo de respondentes, com possível viés de seleção. Também se reconhece a limitação relacionada à distribuição geográfica dos participantes com concentração em determinadas regiões, o que pode reduzir a generalização dos achados para outros contextos institucionais. Apesar de a validade de conteúdo e o processo de resposta constituírem etapas relevantes no desenvolvimento de uma escala, será realizada a validação da estrutura interna para que a EARFAH possa ser aplicada na população a que se destina.

### Contribuições para a área da enfermagem, saúde ou políticas públicas

A construção e a validação da EARFAH representam um avanço para a prática clínica, identificando os pacientes com riscos da ocorrência de flebite e incorporando medidas preventivas. Obter uma escala de medida em enfermagem e saúde, com validade e precisão, é essencial para a segurança e qualidade do cuidado ofertado.

## CONCLUSÕES

A partir da revisão integrativa e da contribuição de especialistas participantes de duas rodadas de avaliação e de enfermeiros que atuaram no processo de resposta, foi possível construir a versão final da EARFAH, composta por três dimensões e 28 itens, que apresenta evidências de validade de conteúdo e de processo de resposta, consistindo na primeira escala brasileira que aborda esse construto na assistência hospitalar.

A EARFAH teve boa aceitação pelos enfermeiros entrevistados no processo de resposta, sendo considerada uma escala de fácil compreensão. No entanto, para sua aplicação, a fim de avaliar o risco de flebite em pacientes adultos hospitalizados, estudos adicionais para avaliação de outras evidências psicométricas devem ainda ser realizados.

## Data Availability

Os dados de pesquisa estão disponíveis no corpo do artigo.
